# The Biostimulant Effect of Hydroalcoholic Extracts of *Sargassum* spp. in Tomato Seedlings under Salt Stress

**DOI:** 10.3390/plants11223180

**Published:** 2022-11-21

**Authors:** Oscar Sariñana-Aldaco, Adalberto Benavides-Mendoza, Armando Robledo-Olivo, Susana González-Morales

**Affiliations:** 1 Program in Protected Agriculture, Universidad Autónoma Agraria Antonio Narro, Saltillo 25315, Coahuila, Mexico; 2 Horticulture Department, Universidad Autónoma Agraria Antonio Narro, Saltillo 25315, Coahuila, Mexico; 3 Food Science & Technology Department, Universidad Autónoma Agraria Antonio Narro, Saltillo 25315, Coahuila, Mexico; 4 National Council for Science and Technology (CONACyT), Universidad Autónoma Agraria Antonio Narro, Saltillo 25315, Coahuila, Mexico

**Keywords:** biostimulation, brown alga, enzymatic and nonenzymatic antioxidants, gene expression, oxidative stress, *Solanum lycopersicum*

## Abstract

Currently, the use of biostimulants in agriculture is a tool for mitigating certain environmental stresses. Brown algae extracts have become one of the most important categories of biostimulants in agriculture, and are derived from the different uses and positive results obtained under optimal and stressful conditions. This study aimed to examine the efficacy of a foliar application of a hydroalcoholic extract of *Sargassum* spp. and two controls (a commercial product based on *Ascophyllum nodosum* and distilled water) with regard to growth, the antioxidant system, and the expression of defense genes in tomato seedlings grown in nonsaline (0 mM NaCl) and saline (100 mM NaCl) conditions. In general, the results show that the *Sargassum* extract increased the growth of the seedlings at the end of the experiment (7.80%) compared to the control; however, under saline conditions, it did not modify the growth. The *Sargassum* extract increased the diameter of the stem at the end of the experiment in unstressed conditions by 14.85% compared to its control and in stressful conditions by 16.04% compared to its control. Regarding the accumulation of total fresh biomass under unstressed conditions, the *Sargassum* extract increased it by 19.25% compared to its control, and the accumulation of total dry biomass increased it by 18.11% compared to its control. Under saline conditions, the total of fresh and dry biomass did not change. Enzymatic and nonenzymatic antioxidants increased with NaCl stress and the application of algal products (*Sargassum* and *A. nodosum*), which was positively related to the expression of the defense genes evaluated. Our results indicate that the use of the hydroalcoholic extract of *Sargassum* spp. modulated different physiological, metabolic, and molecular processes in tomato seedlings, with possible synergistic effects that increased tolerance to salinity.

## 1. Introduction

In general terms, salinity stress is one of the most limiting stresses in crop production, damaging large cultivated areas [1]. This stress negatively influences the absorption and assimilation of water and nutrients, in addition to generating toxicity, which makes it more harmful to crop production [2]. Salinity-induced stress in plants causes a decrease in water potential due to the high concentration of dissolved ions in the soil solution or nutrient solution [3]. Plants subjected to this type of stress suffer physiological, morphological, and biochemical changes such as photosynthesis decrease and growth reduction, stomatal closure, overproduction of reactive oxygen species (ROS), and the production of enzymatic and nonenzymatic antioxidants [3,4]. The overproduction of ROS damages carbohydrates, proteins, lipids, and nucleic acids; however, by producing antioxidants, plants manage to mitigate stress to a certain extent [5,6].

Faced with this situation, traditional measures are taken, such as washing salts from the soil or the use of genetically modified plants; however, these measures are not always kind to the environment [7]. As a response, alternatives have been sought to reduce the adverse effects of salinity stress on crops. One of them is the use of the technique called plant biostimulation. Biostimulation is a phenomenon of the modification of metabolism and gene expression that allows for more efficient use of environmental resources, more significant growth and yield, and a greater tolerance to adverse environmental factors [8]. Biostimulation can be achieved through different environmental stimuli or using biostimulants, e.g., seaweed or botanical extracts. To date, there is no single definition, with unanimous acceptance, of what constitutes a biostimulant. However, among the academic community, there is close agreement on the subject. The most recent definition that is available is Yakhin et al. [9], which mentions that a biostimulant is any formulated product of biological origin that improves plant productivity because of new or emerging properties of the complex of constituents and not only by the essential nutrients, growth regulators or plant protective compounds.

In addition to this, there is the definition of du Jardin [10], which was, at the time, the most accepted definition, and to date, it is the most cited. This definition mentions that a plant biostimulant is any substance or microorganism applied to plants with the aim of enhancing nutrition efficiency, abiotic stress tolerance, and/or crop quality traits, regardless of its nutrient content. The European Union (EU) also has a definition. This definition mentions that a plant biostimulant shall be a fertilizing product, the function of which is to stimulate plant nutrition processes independently of the product’s nutrient content, with the sole aim of improving one or more of the following characteristics of the plant or the plant rhizosphere: (1) nutrient-use efficiency, (2) tolerance to abiotic stress, (3) quality traits, and (4) availability of confined nutrients in the soil or rhizosphere [11].

Seaweed extracts fall within these academic definitions due to the biostimulant power provided by the complex of carbohydrates, amino acids, inorganic compounds, phenolic compounds, carotenoids, and phytohormones that are present in the extracts [12,13,14,15]. In general, the metabolites that are present in the extracts fulfill a protective function in plants, providing greater tolerance to stress [16]. In addition, the extracts are complied with activities that improved the growth and development of plants [14,16].

In the EU there is legislation on biostimulants [11]; however, in other countries, such as Mexico, to date, there is no regulation on them as differentiated products of fertilizers and/or growth regulators. In addition, Mexico has the NOM-182-SSA1-2010 standard [17], which mentions three categories of growth regulators. In category one, which is where algae extracts come in, it is established that they are substances found naturally in plant tissues, whether obtained by extraction, fermentation, synthesis, or other methods. These substances include but are not limited to auxins, gibberellins, cytokinins, ethylene, cofactors, and growth retardants, which, in small quantities, promote, inhibit, or modify the growth and development of plants. According to the above, in Mexico, without legislation on biostimulants, algae extracts could be considered type 1 growth regulators [17].

The use of algae in agriculture has been increasing; however, the most commonly used algae are brown algae due to their higher concentration of metabolites [18]. The brown algae of the genus *Sargassum* today are a pollution problem on the Caribbean coast [19]. Derived from this, *Sargassum* has been given different uses to take advantage of its exaggerated amounts on the coasts. Uses include housing construction, the production of food supplements, the production of cosmetics, and the use of biostimulants in agriculture [20,21,22].

*Sargassum* spp. seaweed extracts (SSE) have shown positive results as inducers of tolerance to abiotic stress. Previously, SSE was shown to improve NaCl tolerance in chickpea crops by improving growth parameters and the enzymatic activity of superoxide dismutase (SOD), ascorbate peroxidase (APX), and peroxidase (POD) [23]. It was also shown that SSE increased the activity of SOD, POD, and catalase (CAT) enzymes and increased the contents of phenols, proline, and total antioxidant activity in barley plants subjected to NaCl stress [24]. In addition, there are reports that mention the regulation of the expression of defense genes in plants, which promotes tolerance to stress [22,25]. In this context, the application of SSE was carried out with the objective of evaluating responses at the agronomic, biochemical, and molecular levels of tomato seedlings subjected to saline stress. The hypothesis is that the SSE applied by foliar spray works as a biostimulant, inducing changes in gene expression and the promotion of antioxidant enzymes or active metabolites to counteract the adverse effects caused by saline stress.

## 2. Results

### 2.1. Seedling Growth and Biomass

Table 1 shows the results of the height, stem diameter, and a number of leaves of tomato seedlings. The results indicate that in plant height under standard growth conditions at 11 DAT, the SSE increased by 17% compared to AC; at 21 DAT, there were no significant differences; and at 31 DAT, the SSE increased by 7.80% compared to AC. For the stressed treatments, there were no differences in any evaluation.

For the stem diameter under standard growth conditions, at 11 DAT, the SSE increased it by 11.32%, at 21 DAT by 12.33%, and at 31 DAT by 14.85%, compared to AC. For the stressed treatments at 11 DAT, the SSE + NaCl increased by 14.76%, at 21 DAT by 14.88%, and at 31 DAT by 16.04%, compared to AC + NaCl.

For the number of leaves under standard growth conditions at 11 DAT, the SSE increased by 23.33%, at 21 DAT by 17.03%, and at 31 DAT by 11.76%, compared to AC. The stressed treatments did not show differences between them.

Table 2 shows the results of fresh and dry biomass of tomato seedlings. In general, the results indicate that for the total fresh biomass under standard growth conditions, the SSE increased by 19.25% compared to AC. Under stress conditions, there were no differences between treatments. For the total dry biomass under standard growth conditions, the SSE increased by 18.11% compared to AC. Under stress conditions, there were no differences between treatments.

**Figure 1:**
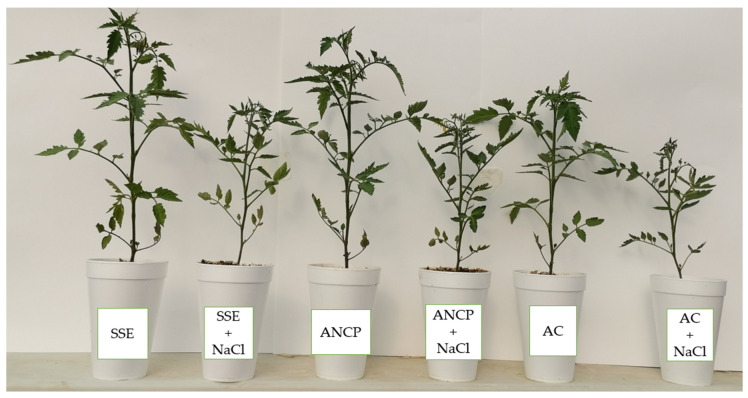
Tomato seedlings in the experimental assay. SSE: *Sargassum* spp. seaweed extracts; ANCP: *A. nodosum* commercial product; AC: Absolute control; NaCl: 100 mM sodium chloride.

Figure 1 shows an image of the seedlings under standard growth conditions and NaCl stress conditions.

### 2.2. Stomatal Conductance of Leaves

**Figure 2:**
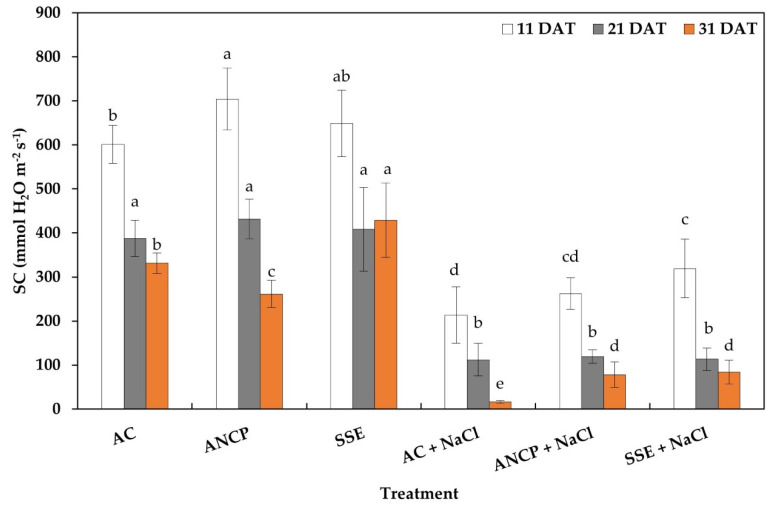
Stomatal conductance in the leaves of tomato seedlings. Different letters within each column indicate significant differences between treatments (LSD, *p* ≤ 0.05). DAT: Days after transplant; SC: Stomatal conductance; AC: Absolute control; ANCP: *A. nodosum* commercial product; SSE: *Sargassum* spp. seaweed extracts; NaCl: 100 mM sodium chloride; *n* = 5; ± bar intervals represent the SD.

The results showed significant differences between treatments, and NaCl stress caused a decrease in stomatal conductance compared to treatments that were not stressed (Figure 2). Regarding the nonstressed treatments, in the first evaluation (11 DAT), ANCP showed the highest conductance, surpassing AC by 17.11%. In the second evaluation (21 DAT), there were no differences, and in the third evaluation (31 DAT), the SSE exceeded the AC by 29.31%. In the stressed treatments, in the first evaluation (11 DAT), SSE + NaCl was statistically higher by 49.70% compared to AC + NaCl. In the second evaluation (21 DAT), there were no differences between the treatments, and in the third evaluation (31 DAT), ANCP + NaCl and SSE + NaCl exceeded AC + NaCl by 3.78 and 4.13 times, respectively.

### 2.3. Photosynthetic Pigments

**Figure 3:**
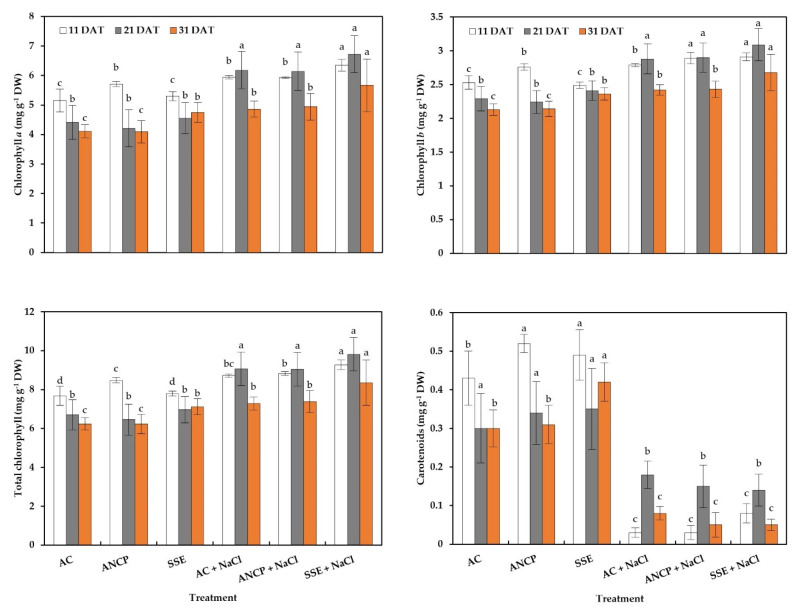
Photosynthetic pigments in the leaves of tomato seedlings. Different letters within each column indicate significant differences between treatments (LSD, *p* ≤ 0.05). DAT: Days after transplant; DW: Dry weight; AC: Absolute control; ANCP: *A. nodosum* commercial product; SSE: *Sargassum* spp. seaweed extracts; NaCl: 100 mM sodium chloride; *n* = 5; ± bar intervals represent the SD.

Figure 3 shows the results for photosynthetic pigments. For chlorophyll *a*, under standard growth conditions in the first sampling (11 DAT), it can be seen that the ANCP exceeded the AC by 10.87%; in the second sampling (21 DAT) there were no differences; and in the third sampling (31 DAT), SSE exceeded the AC by 15.57%. For the stressed treatments, SSE + NaCl, in the first sampling (11 DAT), exceeded AC + NaCl by 6.90%; in the second sampling (21 DAT), there were no differences; and in the third sampling (31 DAT) SSE + NaCl exceeded AC + NaCl by 16.66%.

For chlorophyll *b*, under standard growth conditions in the first sampling (11 DAT), ANCP increased SSE by 10.84%, which was the lowest; in the second sampling (21 DAT), there were no differences; and in the third sampling (31 DAT), SSE exceeded AC by 10.79%. Regarding the stressed treatments in the first sampling (11 DAT), ANCP + NaCl and SSE + NaCl exceeded AC + NaCl by 3.58 and 4.30%, respectively; in the second sampling (21 DAT), there were no differences; and in the third sampling (31 DAT), SSE + NaCl exceeded AC + NaCl by 10.74%.

For total chlorophyll, under standard growth conditions, in the first sampling (11 DAT), ANCP exceeded AC by 10.28%, in the second sampling (21 DAT) there were no differences, and in the third sampling (31 DAT), SSE exceeded AC by 13.94%. For the stressed treatments in the first sampling (11 DAT), SSE + NaCl exceeded AC + NaCl by 6.30%; for the second sampling (21 DAT), there were no differences; and for the third sampling (31 DAT), SSE + NaCl exceeded AC + NaCl by 14.69%.

Regarding carotenoids, it can be seen that stress decreased them compared to treatments that were not stressed, and in the first sampling (11 DAT) under standard growth conditions, ANCP and SSE exceeded AC by 20.93 and 13.95%, respectively. In the second sampling (21 DAT), there were no differences. For the third sampling (31 DAT), the SSE statistically exceeded AC by 40%. The stressed treatments did not present differences in any of the samplings.

### 2.4. Enzymatic Activity and Total Proteins

**Figure 4:**
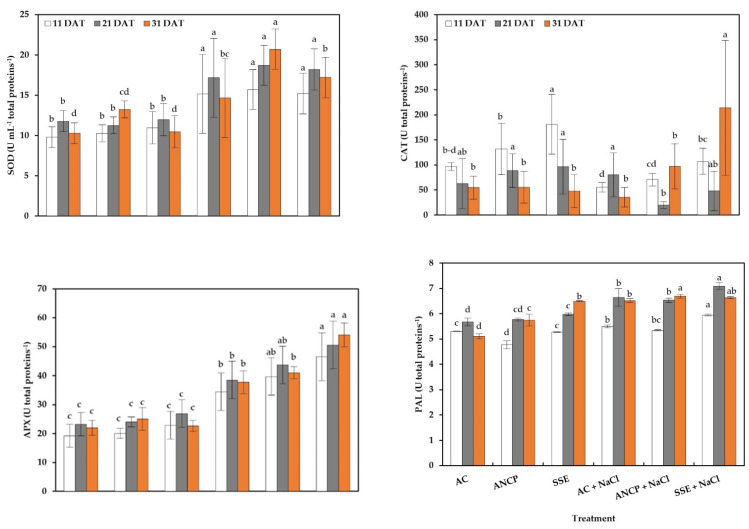

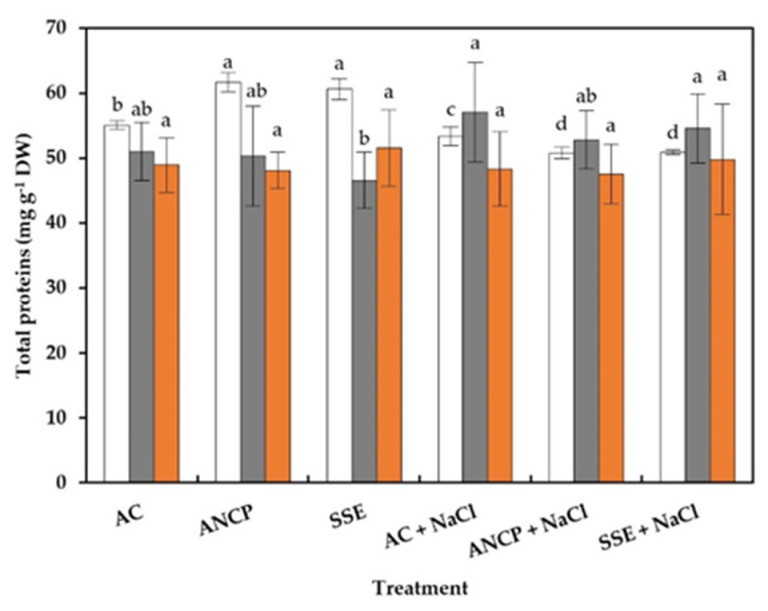
Enzymatic activity and total proteins in the leaves of tomato seedlings. Different letters within each column indicate significant differences between treatments (LSD, *p* ≤ 0.05). DAT: Days after transplant; SOD: Superoxide dismutase; CAT: Catalase; APX: Ascorbate peroxidase; PAL: Phenylalanine ammonium lyase; DW: Dry weight. AC: Absolute control; ANCP: *A. nodosum* commercial product; SSE: *Sargassum* spp. seaweed extracts; NaCl: 100 mM sodium chloride; *n* = 5; ± bar intervals represent the SD.

The results indicate that there were significant differences between treatments (Figure 4). For SOD activity, the stressed treatments at 11, 21, and 31 DAT increased their activity compared to those that were not stressed. In the treatments that were not stressed, there were no significant differences in any sampling. In the first two samplings (11 and 21 DAT), of the stressed treatments, there were no significant differences; only in the last sampling (31 DAT) was ANCP + NaCl the highest compared to AC + NaCl with 41.26%.

In the CAT activity, in the nonstressed treatments, the SSE increased the activity at 11 and 21 DAT by 87.03 and 53.05%, respectively, with respect to the AC; for the 31 DAT, there were no differences. For the stressed treatments, at 11 DAT, the SSE + NaCl increased the activity by 94.05% compared to the AC + NaCl. At 21 DAT, the AC + NaCl exceeded ANCP + NaCl three-fold, which was the lowest, and at 31 DAT, the SSE + NaCl exceeded AC + NaCl by five-fold.

In the APX activity, the stressed treatments and the use of the SSE increased the activity. For the nonstressed treatments, there was no differences in any sampling; however, in the stressed treatments, there were differences. SSE + NaCl increased the activity by 35.01% (11 DAT), 31.37% (21 DAT), and 43.10% (31 DAT) compared to AC + NaCl.

In the activity of phenylalanine ammonia-lyase (PAL), it can be seen in the treatments that were not stressed, ANCP showed lower activity than AC and SSE at 11 DAT. At 21 and 31 DAT, the SSE exceeded the AC by 5.46 and 7.25%, respectively. Regarding the stressed treatments at 11 and 21 DAT, SSE + NaCl exceeded AC + NaCl by 8.19 and 6.77%, respectively. At 31 DAT, ANCP + NaCl exceeded AC + NaCl by 2.76%.

For total proteins, it can be seen that for the treatments with and without stress, there were only differences in the first sampling (11 DAT). The ANCP and SSE exceeded the AC by 12.04 and 10.12%, respectively. AC + NaCl exceeded ANCP + NaCl and SSE + NaCl by 5.20 and 4.91%, respectively.

### 2.5. Hydrophilic Antioxidants and Antioxidant Capacity

**Figure 5:**
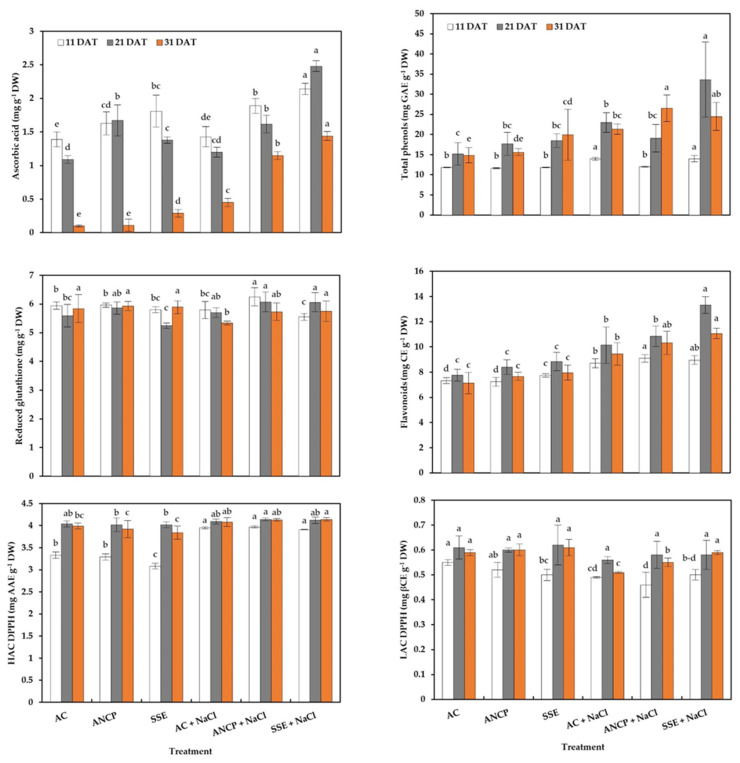
Hydrophilic antioxidants and antioxidant capacity in the leaves of tomato seedlings. Different letters within each column indicate significant differences between treatments (LSD, *p* ≤ 0.05). DAT: Days after transplant; DW: Dry weight; GAE: Gallic acid equivalents; CE: Catechin equivalents; HAC: Hydrophilic antioxidant capacity; DPPH: 2,2-diphenyl-1-picrilhidrazilo; AAE: Ascorbic acid equivalents; LAC: Lipophilic antioxidants capacity; βCE: β-carotene equivalents; AC: Absolute control; ANCP: *A. nodosum* commercial product; SSE: *Sargassum* spp. seaweed extracts; NaCl: 100 mM sodium chloride; *n* = 5 except ascorbic acid (*n* = 4); ± bar intervals represent the SD.

Regarding nonenzymatic antioxidants and antioxidant capacity, some differences between treatments were observed (Figure 5). For ascorbic acid, in the nonstressed treatments, it was observed that at 11 DAT, the SSE exceeded the AC by 7.10%; at 21 DAT, the ANCP exceeded the AC by 53.21%; and at 31 DAT, the SSE exceeded the AC 1.9-fold. Regarding the stressed treatments, SSE + NaCl increased the content by 49.65% (11 DAT), 106% (21 DAT) and 2.2-fold (31 DAT), compared to AC + NaCl.

For the content of reduced glutathione (GSH), in the nonstressed treatments, no significant differences were observed compared to the AC in any sampling. For the stressed treatments at 11 DAT, ANCP + NaCl exceeded SSE + NaCl by 12.61%, which was the lowest. For 21 DAT, there were no differences, and for 31 DAT, the SSE + NaCl exceeded the AC + NaCl by 7.67%.

For total phenols, in the nonstressed treatments, differences were observed only at 31 DAT, with SSE exceeding AC by 34.41%. Regarding the stressed treatments at 11 DAT, ANCP + NaCl showed a decrease with respect to AC + NaCl and SSE + NaCl; for 21 DAT, SSE + NaCl exceeded ANCP + NaCl by 76.31%, which was the lowest. At 31 DAT, ANCP + NaCl exceeded AC + NaCl by 24.12%.

For flavonoids, in the nonstressed treatments, there was only a difference at 11 DAT, with the SSE exceeding the ANCP by 6.90%, which was the lowest. For the stressed treatments at 11 DAT, ANCP + NaCl exceeded AC + NaCl by 4.48%; at 21 and 31 DAT, SSE + NaCl increased the concentration by 31.39 and 17.16%, respectively, compared to AC + NaCl.

For the hydrophilic antioxidant capacity, there were only differences at 11 DAT of the unstressed treatments, with AC and ANCP exceeding SSE by 8.11 and 6.81%, respectively.

For the lipophilic antioxidant capacity, in the nonstressed treatments, there was only a difference at 11 DAT, where the SSE was lower than the AC. Regarding the stressed treatments, only at 31 DAT were differences shown, with SSE + NaCl exceeding AC + NaCl by 15.68%.

### 2.6. Proline and Hydrogen Peroxide

**Figure 6:**
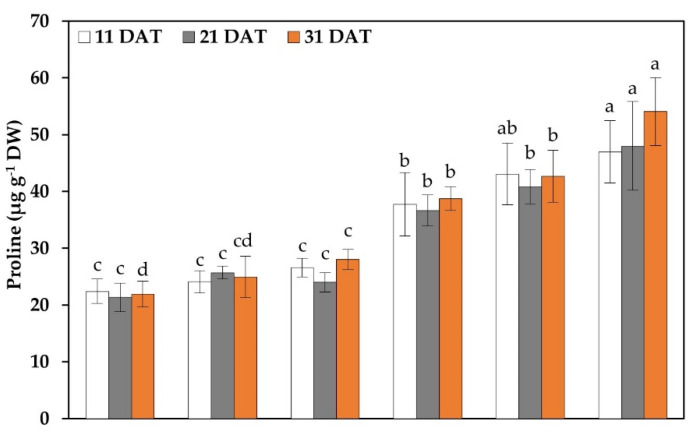

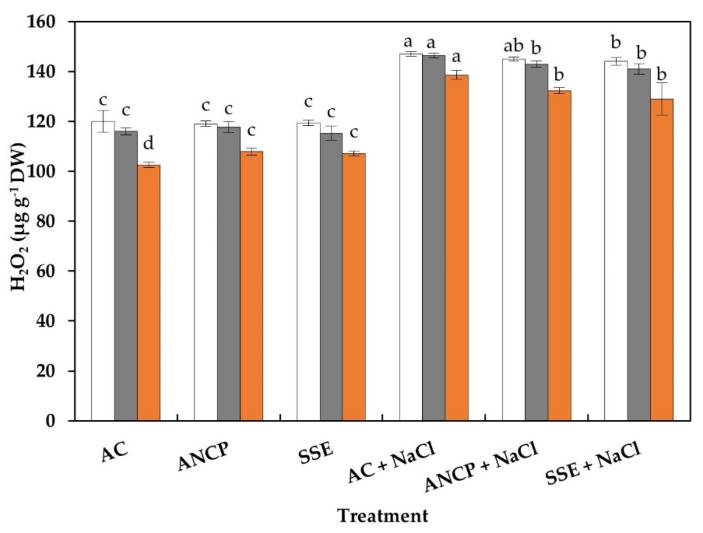
Proline and hydrogen peroxide in the leaves of tomato seedlings. Different letters within each column indicate significant differences between treatments. (LSD, *p* ≤ 0.05). DAT: Days after transplant; DW: Dry weight; AC: Absolute control; ANCP: *A. nodosum* commercial product; SSE: *Sargassum* spp. seaweed extracts; NaCl: 100 mM sodium chloride; *n* = 5; ± bar intervals represent the SD.

The results showed significant differences in the concentrations of hydrogen peroxide and proline between treatments (Figure 6). For proline in the treatments without stress, there were only differences at 31 DAT, where the SSE exceeded the AC by 27.73%. For the stressed treatments, the SSE + NaCl exceeded the AC + NaCl by 24.50% (11 DAT), 31.07% (21 DAT), and 39.64% (31 DAT).

For hydrogen peroxide, in the treatments without stress, there was only a difference at 31 DAT, where ANCP and SSE exceeded the AC by 5.26 and 4.55%, respectively. For the stressed treatments, AC + NaCl at 11 DAT increased its concentration compared to SSE + NaCl by 2.02%, and at 21 and 31 DAT, AC + NaCl exceeded ANCP + NaCl and SSE + NaCl.

### 2.7. Expression of Defense Genes

**Figure 7:**
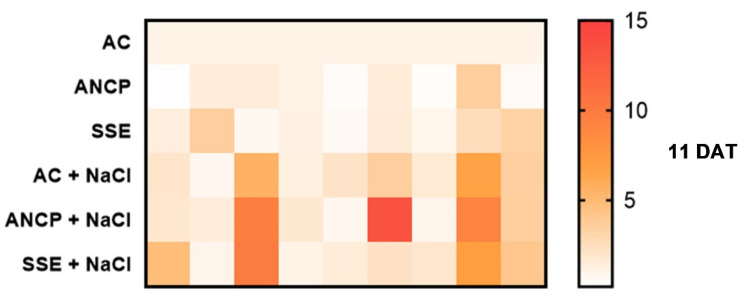

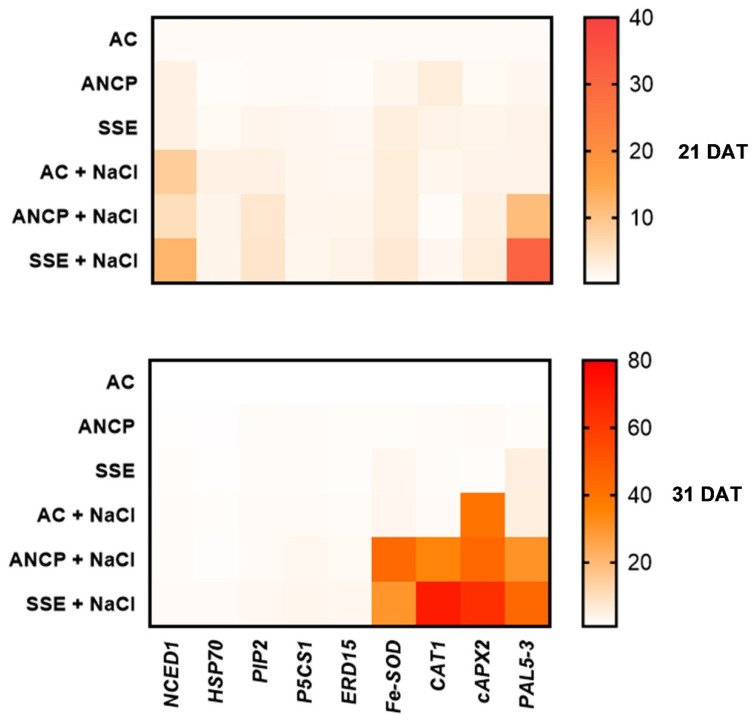
Heatmap of the relative expression of defense genes in leaves of tomato seedlings. DAT: Days after transplant; AC: Absolute control; ANCP: *A*. *nodosum* commercial product; SSE: *Sargassum* spp. seaweed extracts; NaCl: 100 mM sodium chloride; *n* = 4. The AC represents a constant value of 1 at the expression level.

The results indicate that the use of algae products (SSE and ANCP) and NaCl stress induced the expression of the genes evaluated in the three samplings (Figure 7). In unstressed treatments, SSE increased the expression of the *NCED1* gene by 1.35-fold (11 DAT), 2.36-fold (21 DAT), and 1.68-fold (31 DAT) compared to AC. In stressed treatments, the SSE + NaCl, increased the expression of the *NCED1* gene by 4.77-fold (11 DAT), 12.35-fold (21 DAT), and 2.24-fold (31 DAT) compared to AC.

For the *HSP70* gene, in the treatments without stress, the SSE increased its expression 3.54-fold (11 DAT), 1.08-fold (21 DAT), and 1.17-fold (31 DAT), compared to AC. For the stressed treatments, ANCP + NaCl increased its expression 1.4-fold at 11 DAT, AC + NaCl increased by 2.37-fold at 21 DAT, and SSE + NaCl increased by 2.13-fold at 31 DAT, compared to AC.

For the *PIP2* gene, in treatments without stress, ANCP increased its expression 1.46 and 2.04-fold at 11 and 31 DAT, compared to AC. At 21 DAT, the SSE increased its expression 1.69-fold, compared to the AC. In the stressed treatments, SSE + NaCl increased its expression 9.91-fold (11 DAT), 4.35-fold (21 DAT), and 2.62-fold (31 DAT), compared to AC.

For the *P5CS1* gene, in the unstressed treatments, SSE increased its expression at 11 and 21 DAT (1.15 and 1.45-fold, respectively), as well as ANCP (1.947-fold) at 31 DAT, compared to AC. At 11 and 21 DAT, ANCP + NaCl increased its expression (1.78 and 1.69-fold, respectively), as well as SSE + NaCl (3.27-fold) at 31 DAT, compared to AC.

For the *ERD15* gene, in treatments without stress, at 11 DAT, ANCP and SSE repressed their expression (0.41 and 0.58-fold, respectively); at 21 and 31 DAT, SSE increased its expression 1.24- and 1.74-fold, respectively, compared to AC. In stress treatments at 11 DAT, AC + NaCl increased its expression 2.08-fold; at 21 DAT, ANCP + NaCl increased its expression 1.69-fold; and at 31 DAT, SSE + NaCl increased its expression 2.94-fold, compared to AC.

The *Fe-SOD* gene showed an increase in expression in the stressed treatments. In treatments without stress, SSE had the highest expression by 1.53, 2.7, and 2.98-fold, respectively, for 11, 21, and 31 DAT, compared to AC. At 11 DAT, ANCP + NaCl increased its expression by 13.41-fold; at 21 DAT, SSE + NaCl increased its expression by 3.7-fold; and at 31 DAT, ANCP + NaCl increased its expression by 43.38-fold, compared to AC.

For the *CAT1* gene, in treatments without stress at 11 DAT, ANCP and SSE repressed their expression (0.38 and 0.82-fold, respectively); at 21 DAT, ANCP increased its expression 3.06-fold; and at 31 DAT, SSE increased its expression 2.07-fold, compared to AC. In stress treatments, at 11 DAT, SSE + NaCl increased its expression 1.85-fold; at 21 DAT, AC + NaCl increased its expression 1.54-fold; and at 31 DAT, SSE + NaCl increased its expression 70.5-fold, compared to AC.

The *cAPX2* gene showed an increase in expression in the three samplings for the stressed treatments. At 11 DAT, ANCP + NaCl increased its expression 9.25-fold; and at 21 and 31 DAT, SSE + NaCl increased its expression by 3.07-fold and 63.64-fold, respectively, compared to AC. For treatments without stress, ANCP increased its expression at 11 and 31 DAT (3.58 and 2.29-fold, respectively) compared to AC. At 21 DAT, the SSE increased its expression 1.82-fold, compared to the AC.

The expression of the *PAL5-3* gene with respect to the stressed treatments was increased compared to the nonstressed treatments. For the stressed treatments, SSE + NaCl increased its expression by 4.1-fold (11 DAT), 31.07-fold (21 DAT), and 44.17-fold (31 DAT), compared to AC. For unstressed treatments, SSE increased its expression 3.26-fold (11 DAT), 2.14-fold (21 DAT), and 5.12-fold (31 DAT) compared to AC.

## 3. Discussion

### 3.1. Seedling Biomass and Growth Parameters

Crops that grow in high concentrations of salts tend to reduce their development and growth; therefore, production is reduced [7,26,27]. Salt stress in plants causes osmotic stress, which triggers plants to consume extra energy to absorb water and nutrients [28,29]. This effect is analogous to that produced by water stress, when the plant increases the respiration rate to produce more energy to absorb water [30,31]. Derived from this extra energy consumption, plants reduce growth and biomass accumulation, as observed in the results of this study; however, the use of SSE improved these parameters to a certain extent. There are different investigations that show that SSE has a positive influence on aspects related to the growth and biomass of plants. Abdel Latef et al. [23] reported that the extracts of *Sargassum muticum* (1%) caused a positive effect on growth variables and on the biomass of the chickpea crop under stress conditions by NaCl (50 and 150 mM). On the other hand, Gharib et al. [32] indicated that the foliar application of extracts of *Sargassum latifolium* (0.4%) increased the growth parameters (height) and biomass (fresh and dry weights of the shoot) of rosemary plants under salt stress (100 mM NaCl). In addition, they also mention that the extracts at all of the concentrations used improved the growth of the plants in conditions without stress.

The positive effects are induced due to the biostimulant compounds that SSE contains (phytohormones, carbohydrates, proteins, amino acids, and GSH), which help the plant in the absorption and translocation of nutrients under NaCl stress conditions, and in the same way, stimulate the C and N metabolism [14,22,33,34,35]. On the other hand, auxins, mainly indole-3-acetic acid (IAA), control physiological processes in plants, such as cell elongation and division [36]. Cytokinins, mainly trans-zeatin (tZ), activate lateral bud growth and stimulate nutrient translocation [37,38]. Carbohydrates facilitate the assimilation and transport of mineral elements and are the main source of energy in plants [39,40]. Nitrogenous metabolites (proteins, amino acids, and GSH), being a source of nitrogen and hormonal precursors, positively modify metabolism, which improves the growth and accumulation of biomass in plants [41,42,43].

### 3.2. Stomatal Conductance

This variable indicates the rate of water vapor diffusion via the stomata from the mesophyll to the atmosphere [44]. This variable also works as an indicator when plants are under water deficit [45]. As mentioned above, NaCl in plants causes water stress that deprives them of this resource so plants tend to close their stomata and reduce stomatal conductance to avoid water loss as much as possible [46,47]. The results of the present investigation show that the treatments without stress showed a greater stomatal conductance. Similar results were reported by Miceli et al. [48], indicating that the use of extracts of *Ecklonia maxima* in lettuce cultivation increased stomatal conductance at all concentrations (1, 2, and 4 mL L^−1^) compared to the control. The results are because brown algae extracts contain osmolytes such as proline, glycine betaine, and carbohydrates, compounds that allow for osmotic adjustment and that facilitate the absorption of water and nutrients by plants [49,50,51]. These osmolytes can stabilize the hydration sphere of proteins and membranes and reduce the water potential in cells under conditions of osmotic stress [52], which can explain the improvement in stomatal conductance. It is important to mention that at higher rates of stomatal conductance, biomass accumulation is improved, to a certain extent, since photosynthetic activity is increased by greater carbon dioxide (CO_2_) uptake [45].

### 3.3. Photosynthetic Pigments

Chlorophylls are the most important pigments that plants have since they control photosynthetic activity by capturing solar light energy [53]. However, chlorophylls are not the only pigments that are involved in the photosynthetic process, since carotenoids also play an important role by having photoprotective properties and capturing light in spectral regions that are not covered by chlorophylls, which broadens the range of wavelengths in which light can be used in photosynthesis [54,55]. The results of the present investigation showed that with NaCl stress and the use of SSE, the concentration of chlorophylls increased, but that of carotenoids decreased. Similar results were shown by Hernández-Hernández et al. [56], indicating that stress by NaCl (100 mM) in tomato leaves increased the concentration of chlorophylls compared to treatments that were not stressed. They also indicated that stress increased the concentration of carotenoids, which differs from the present experiment. Similarly, Morales-Espinoza et al. [57] indicated that stress by NaCl (50 mM) increased the concentration of chlorophylls in tomato leaves, compared to the control treatment. Zou et al. [16] indicated that stress by NaCl (150 mM) caused a significant decrease in the contents of chlorophylls *a* and *b* in wheat seedlings; however, with the use of polysaccharides derived from the brown algae *Lessonia nigrescens,* the content was increased. Vijayanand et al. [58] mentioned that the foliar application of *Sargassum wightii* extracts (1.5%) improved the contents of chlorophylls *a* and *b* in bean plants.

The results in the first instance are caused by NaCl stress, which causes plants to make the photosynthetic process more efficient, increasing the concentration of chlorophylls, up to a certain point, due to the limitation of water and nutrients [59]. The photosynthetic capacity of leaves is closely related to the N content since the proteins that make up the thylakoids and those that function in the Calvin cycle represent the majority of the foliar N [60,61]. In this way, SSE, being a source of nitrogenous metabolites (proteins, amino acids, and GSH), can increase the concentration of pigments. In the same way, SSE contains glycine betaine, which is a quaternary amine; in addition to being a source of N, its main role is involved in photosynthetic activity since it protects chlorophylls from oxidation [51,62]. Regarding carotenoids, the results are attributed to the fact that pigments are responsible for the detoxification of free radicals and the dissipation of excess energy, for which, when there is severe stress, they will be the first pigments to oxidize, protecting the chlorophylls [54].

### 3.4. Enzymatic Activity, Hydrophilic Antioxidants, and Antioxidant Capacity

Plants are sessile organisms, and to survive and reproduce, they not only need to grow and develop but also must continuously tolerate and adapt to stress (pathogens, drought, extreme temperatures, and salinity, among others) [63,64,65]. In this situation, plants activate enzymatic and nonenzymatic antioxidant systems, which are responsible for protecting cells against ROS produced by stress [63,66,67]. The enzyme antioxidant system is the first line of defense in plants that neutralizes ROS; these enzymes include SOD, CAT, APX, and glutathione peroxidase (GPX) [67,68]. There are other enzymes that are not classified as antioxidants; however, they are indirectly involved, and one of them is PAL, which catalyzes the first reaction of the phenylpropanoid pathway, compounds with high antioxidant capacities [69]. Nonenzymatic antioxidants, whether hydrophilic (phenols, GSH, and ascorbic acid) or lipophilic (carotenoids), are the second line of defense and are responsible for inhibiting the production of damage caused by the oxidative reaction [67,70].

The results of the present investigation show that with stress and brown algae products (SSE and ANCP), it was possible to increase the activity of the enzymes, the accumulation of hydrophilic antioxidants, and the hydrophilic antioxidant capacity. Sofy et al. [24] mentioned that the use of extracts of *S. latifolium* (30%) in barley plants under saline conditions (NaCl, 75 and 150 mM) increased the activities of the SOD, CAT, and POD enzymes. Similarly, Elansary et al. [71] reported an increase in APX activity with the use of *A. nodosum* extracts (5 and 7 mL L^−1^) in *Paspalum vaginatum* under NaCl stress conditions (49.7 dS m^−1^). Aitouguinane et al. [72] indicated that the use of alginates and oligoalginates, isolated from the brown alga *Bifurcaria bifurcata,* in tomato seedlings increased the activity of the PAL enzyme, which was positively correlated with the increase in total phenols, which coincides with the present study.

Similar results are reported by Hashem et al. [73], who mention that the use of the brown alga *Cystoseira* Spp. in the canola crop under NaCl stress conditions (75 and 150 mM) increased the contents of phenols, flavonoids, anthocyanins, and DPPH antioxidant capacity. Kumari et al. [74] mentioned that the foliar, drenching, and combined application of extracts (10%) of *Sargassum johnstonii* increased the contents of phenols, ascorbic acid, and lycopene in tomato leaves and fruits.

NaCl stress, by causing water and nutrient deficits, and toxicity, leads to the overproduction of ROS in plant cells, and therefore, to the activation of the enzymatic and nonenzymatic antioxidant system that detoxifies the cells of ROS [7,66,75,76]. Brown algae extracts also activate the defense system of plants. Shukla et al. [77] indicated that the metabolites present in the extracts induce the increase and synthesis of antioxidants, both enzymatic and nonenzymatic. In the present investigation, stress caused a decrease in carotenoids, which correlates with a lower lipophilic antioxidant capacity; however, the use of SSE in stress situations helped to improve the antioxidant capacity to a certain extent, compared to AC + NaCl.

### 3.5. Total Protein, Proline, and Hydrogen Peroxide

Proteins are of vast importance in plant metabolism. There are catalytic, transport, structural, defense, and reserve proteins, which are involved in all of the metabolic processes of plants [78,79,80,81,82]. In the present study, it was observed that in the first sampling (11 DAT), there were significant differences. Nonstress treatments, specifically SSE and ANCP, increased the protein concentration compared to the stress treatments. Sofy et al. [24] reported that with the application of NaCl at 75 and 150 mM in barley culture, the protein content decreased, but with the application of extracts of *S. latifolium,* it increased substantially. The results are attributed to the products of algae since they contain nitrogenous metabolites such as proteins; in addition, they stimulate the synthesis of these in plants [83]. ROS are generated due to stress and cause the oxidation of lipids, nucleic acids, and proteins, which is why they decrease with NaCl stress [84].

Proline is an amino acid; under normal conditions, it is found in low quantities in plants; however, under stress conditions, its concentration increases to act as an osmotic agent, protecting cells from dehydration and oxidative stress [85,86]. In this study, under NaCl stress conditions and with the use of SSE, the proline concentration increased. Similar results were shown by Zou et al. [87], indicating that with stress by NaCl at 120 mM and the use of fucoidan extracted from the brown algae *Macrocystis pyrifera*, the proline content increased compared to the control in wheat seedlings. The results are a product of the stress caused by the use of the SSE. With osmotic stress, plants increase their concentration of osmolytes, such as proline, glycine betaine, and carbohydrates, compounds that allow for osmotic adjustment and that facilitate the absorption of water and nutrients by plants [50,88,89]. These osmolytes are also involved in maintaining protein structure under stressful conditions [90]. As mentioned above, SSE contains this type of osmolyte, which is why the proline concentration was increased.

Plant metabolism, when subjected to stress, produces ROS, including H_2_O_2_, a toxic species in high concentrations, which in turn is reduced to water and O_2_ by the enzymes CAT, APX, and GPX. In this study, CAT and APX activity were increased with the application of SSE in tomato seedlings [68]. The enzymes APX and GPX use ascorbate and GSH, respectively, as electron donors, to carry out the reduction of H_2_O_2_ [68]. This study shows that stress increased the concentration of H_2_O_2_; however, the use of alga products reduced it to a certain extent. Zou et al. [87] showed similar results, where NaCl stress increased H_2_O_2_ in wheat seedlings, but the use of fucoidan extracted from the algae *M. pyrifera* reduced it considerably. As mentioned above, H_2_O_2_ at high concentrations is toxic; however, being below the threshold level, it is important in plant cell metabolism by functioning as a signaling molecule in different processes, both under stress conditions and without stress [91]. The reduction in H_2_O_2_ in the stressed treatments and with the application of SSE is probably because SSE helps plants synthesize antioxidant compounds that decrease or neutralize ROS.

### 3.6. Expression of Defense Genes

The plant defense system includes enzymatic and nonenzymatic systems, but to achieve this, processes occur that are of great importance and that have not been explored in detail. These processes are linked to the expression of genes that code for proteins involved in the defense system [92]. Plants, being sessile organisms, have the ability to defend themselves against environmental stresses through the expression of defense genes, which will make them have an arsenal of effector biomolecules; however, there are different alternatives, such as genetic engineering, that allow them to always keep certain genes of interest active (gene overexpression). However, there are also other alternatives, such as the use of biostimulants, specifically the use of brown alga extracts that have the ability to activate the plant defense system through the differential expression of genes [25,93,94].

In the present experiment, in the three samplings, there was differential expression of all of the genes studied, especially with the treatments under stress by NaCl and the use of SSE. Drira et al. [95] mention in their study that with the application of *Padina pavonica* extracts in *Arabidopsis thaliana* under NaCl stress, the genes *SOD*, *CAT*, *POD*, and *P5CS* were expressed, which conferred a certain tolerance to the plants. In the same way, Zou et al. [16] indicated that the use of polysaccharides derived from *L. nigrescens* in wheat seedlings stressed by NaCl (150 mM) produced an overexpression of the genes that code for high-affinity potassium transporters (*HKT2;1*) and membrane antiporters Na^+^/H^+^ (*SOS1* and *NHX2*). Al-Ghamdi y Elansary [96] applied extracts of *A. nodosum* in asparagus under saline irrigation (NaCl) and indicated that the *PIP1*, *P5CS1*, *APX1*, and *GPX3* genes were differentially expressed.

The results are the product of the stress caused by NaCl and, consequently, by the application of the algae products. The metabolites present in the algal products (carbohydrates, proteins, amino acids, carotenoids, phytohormones, vitamins, and phenolic compounds) bind to specific sensors in the cell membranes, which trigger a series of signals that lead to the expression of defense genes, which, in turn, code for proteins that directly or indirectly mitigate the adverse effects of stress [77,97]. Cell membranes also have sodium sensors that generate the expression of genes that code for antioxidant and transporter proteins, with the ability to exclude salts from cells or to store them in central vacuoles [97,98].

Among the genes evaluated, *Fe-SOD*, *CAT1*, *cAPX2*, and *PAL5-3,* the functions of the metabolites that they encode have been explained previously, but not for the rest. *P5CS1* encodes the enzyme ∆1-pyrroline-5-carboxylate synthase 1, which catalyzes the synthesis of the amino acid proline [99]. *NCED1* encodes the enzyme 9-cis-expoxycarotenoid dioxygenase 1, which catalyzes the synthesis of abscisic acid in chloroplasts. This phytohormone regulates various processes in plants, including stomatal closure under conditions of osmotic stress [100]. *HSP70* encodes a heat shock protein that protects other biomolecules from denaturation or misfolding under stressful conditions [101]. *PIP2* codes for aquaporins that are responsible for regulating the movement of water across cell membranes under conditions of osmotic stress [102]. *ERD15* encodes proteins involved in the stabilization and renaturation of biomolecules affected by biotic and abiotic stress [103].

It is important to mention that *Sargassum* algae play an important role in the ecological balance of the oceans, and consequently, due to its massive accumulations on the coasts, it is already an environmental problem that has caused the death of marine species such as turtles and fish. However, different uses are being given to *Sargassum* (construction, food supplements, cosmetics, and agricultural products), which somehow mitigate its environmental impact. For this reason, it is important to take advantage of this type of natural resource in agricultural activities.

## 4. Materials and Methods

### 4.1. Plant Material and Experimental Conditions

CID F1 hybrid tomato seeds (Harris Moran Seed Company, Modesto, CA, USA; saladette type and indeterminate growth) were sown in polystyrene trays with a mixture of peat moss and perlite (1:1 *v*/*v*). The experiment was established in a greenhouse covered with polyethylene in the Department of Horticulture of the Universidad Autónoma Agraria Antonio Narro (Saltillo, México). The average temperature was 28 °C, and 50 to 60% relative humidity was used. The seedlings were grown for 30 days until they developed four true leaves. Subsequently, they were transplanted into 1 L containers with the same ratio of substrate used for sowing. The irrigation system was manual, giving one irrigation per day at field capacity. Plant nutrition was performed with a 25% Steiner nutrient solution [104].

### 4.2. Treatments

A 1.5% hydroalcoholic SSE was used, produced by a batch reactor under the conditions of 160 °C, 30 min, and 50% ethanol. The ratio used for the extraction was 1:20 (1 g of algae and 20 mL of 50% ethanol). The dose of SSE used was in accordance with research carried out by Ramya et al. [105], Kasim et al. [106], and Ramya et al. [107], and the extraction condition was selected based on the results obtained in a preliminary test established by Sariñana-Aldaco et al. [14]. Biochemical characterization was performed on the SSE, which is shown in Table 3.

**Figure 8:**
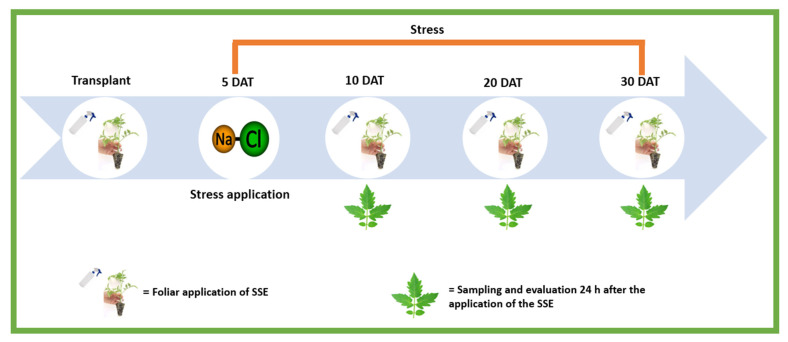
Schematic representation of the experiment. SSE: *Sargassum* spp. seaweed extracts; DAT: Days after transplant.

The treatments applied were the following: (1) control with distilled water (AC), (2) *A. nodosum* commercial product (BYOALG^®^) at 0.13%, based on the specifications for use (ANCP), (3) application of the *Sargassum* spp. seaweed extract (SSE), (4) AC and 100 mM NaCl (AC + NaCl), (5) ANCP and 100 mM NaCl (ANCP + NaCl), and (6) SSE and 100 mM NaCl (SSE + NaCl), giving a total of six treatments. Foliar applications for the algae extracts were applied, a manual sprinkler was used, and the plants were sprayed to the point of dripping. The applications were made every 10 days from the transplant, accumulating four applications during the experiment, which lasted 31 days (Figure 8). The applications were made between 08:00 and 10:00 h. To prevent spraying from neighboring treatments, a flexible plastic barrier was used to isolate the application space. NaCl stress was applied from the fifth day after transplantation (DAT) in the nutrient solution until the end of the experiment.

### 4.3. Sampling and Evaluations

Three destructive samplings and three evaluations were carried out 24 h after the second application of the extracts (11 DAT), 24 h after the third application of the extracts (21 DAT), and 24 h after the fourth application of the extracts (31 DAT) (Figure 8). Destructive sampling consisted of five plants per treatment to determine stress indicator metabolites, and four plants for the expression of defense genes. These samplings were carried out by removing all the leaves of the seedlings, immediately freezing them with liquid nitrogen, and storing them in an ultrafreezer at −80 °C. The evaluations consisted of five plants per treatment that were chosen to measure plant height, number of leaves, stem diameter, and stomatal conductance of the leaves (SC-1 Leaf porometer, ICT International). At the end of the experiment, the fresh and dry biomasses of the seedlings were quantified.

### 4.4. Biochemical Analyses

To determine the stress indicator metabolites in the leaves of the tomato seedlings, the frozen tissue was lyophilized and macerated with a hand mortar to later perform the extractions and quantifications according to the methodologies used.

The contents of photosynthetic pigments were determined according to the Wellburn method [108], with modifications. Total protein was determined using the method described by Bradford [109]. The enzymatic activity of SOD (EC 1.15.1.1) was determined using a Cayman^®^ 7060002 commercial kit (Cayman Chemical Company, Ann Arbor, MI, USA) [27], and the results are expressed in U mL^−1^ per total protein. One unit of SOD is defined as the amount of enzyme required to exhibit a 50% dismutation of the superoxide radical. The enzyme activity of CAT (EC 1.11.1.6) was determined as described in Dhindsa et al. [110], and their results are reported as U (consumption in mM hydrogen peroxide per minute) per total protein. APX activity (EC 1.11.1.11) was determined as described in Nakano and Asada [111], and the results are reported as U (µmol of ascorbic acid oxidized per minute) per total protein. The activity of PAL (EC 4.3.1.5) was determined according to that established by Sykłowska-Baranek et al. [112], and their results are reported as U (production in µmol of trans-cinnamic acid per minute) per total protein.

Ascorbic acid was determined via high-performance liquid chromatography (HPLC) (HPLC VARIAN 920LC) using the method described by Nour et al. [113]. GSH was quantified using the method of Xue et al. [114] via the reaction of 5,5-dithio-bis-2 nitrobenzoic acid (DTNB). Total phenols were determined using the method of Singleton et al. [115] via the Folin–Ciocalteu reaction. Flavonoids were quantified via the aluminum chloride method, as described by Zhishen et al. [116]. Hydrophilic and lipophilic antioxidant capacities were determined using the DPPH radical, as described by Brand-Williams et al. [117], with some modifications. The hydrophilic compounds were extracted with 100% methanol, and the lipophilic compounds were extracted with hexane-acetone (1:1 *v*/*v*).

Free proline content was quantified using the method described by Bates [118]. H_2_O_2_ was quantified using the methodology described by Antoniou et al. [119], using potassium iodide as the reaction agent.

The amino acid content of SSE was determined according to Yemm and Cocking [120]. IAA, tZ, and monosaccharides of SSE were quantified by HPLC using the methodologies of Bosco et al. [121], Rivas-Martínez et al. [122], and Rodríguez-Jasso et al. [123], respectively. The rest of the metabolites of SSE were determined according to the methodologies described above.

For the determination of photosynthetic pigments, proteins, enzymatic activity, GSH, total phenols, flavonoids, antioxidant capacity, proline, H_2_O_2_, and amino acids, a UV-Vis spectrophotometer was used (Thermo Scientific Model G10S, Waltham, MA, USA).

### 4.5. Real-Time Reverse Transcription PCR

TRI reagent (TRI Reagent^®^) was used to extract RNA from the leaves of tomato seedlings, which were subsequently purified with chloroform and precipitated with isopropanol, as described by Cui et al. [124]. The RNA was treated with DNase I (Sigma-Aldrich, Burlington, MA, USA) and quantified using a UV-Vis spectrophotometer with the *A*_260_/*A*_280_ nm ratio, and the quality was determined by denaturing electrophoresis. cDNA synthesis was performed using a commercial Bioline kit (SensiFAST cDNA Synthesis Kit). The primers were acting as an endogenous gene (*ACT*) and nine study genes: *NCED1* (9-cis-expoxycarotenoid dioxygenase 1), *HSP70* (heat shock protein), *PIP2* (aquaporin), *P5CS1* (delta1-pyrroline-5-carboxylate synthase 1), *ERD15* (protein of late embryogenesis), *Fe-SOD* (iron superoxide dismutase), *CAT1* (catalase), *cAPX2* (cytosolic ascorbate peroxidase), and *PAL5-3* (phenylalanine ammonia lyase). The primers were designed using Primer BLAST software (National Center for Biotechnology Information NCBI, Bethesda, Rockville, MD, USA) and Oligoanalyzer 3.1 (Integrated DNA Technologies IDT, Coralville, IA, USA), except for *ERD15*, *Fe-SOD*, and *cAPX2*, which were obtained from Ziaf et al. [103] and Mascia et al. [125], respectively. The sequences of the primers used are described in Table 4.

Real-time PCRs were performed on Applied Biosystems StepOne™ Equipment version 2.3 (Thermo Fisher Scientific, Waltham, MA, USA) using the ∆∆Ct method, measuring the fluorescence intensity of SYBR™ Select Master Mix (Applied Biosystems, Foster City, CA, USA). PCR was performed in a volume of 20 µL for all genes (10 µL Master Mix, 1 µL of cDNA, the concentration of primers, and nuclease-free water). For the actin gene, the concentration of the forward primer was 72 nM, and 60 nM was used for the reverse primer. For the *NCED1*, *ERD15*, *cAPX2*, and *Fe-SOD* genes, the concentrations of the primers were 300 nM equimolar. For the *HSP70* gene, the concentration of the forward primer was 80 nM, and that of the reverse primer was 100 nM. For the *PIP2* and *P5CS1* genes, the primer concentration was 100 nM equimolar. For the *CAT1* gene, the primer concentration was 200 nM equimolar. For the *PAL5-3* gene, the concentration of the forward primer was 150 nM, and 100 nM for the reverse primer. Real-time PCR was run under the following conditions: 10 min at 95 °C and PCR (40 cycles): 15 s at 95 °C and 1 min at 60 °C.

### 4.6. Experimental Design and Data Analysis

A completely randomized design with a 3 × 2 factorial arrangement was used, considering 20 repetitions per treatment. An analysis of variance and Fisher’s LSD test of means (*p* ≤ 0.05) were performed. All statistical procedures were performed using Infostat software (v2020). Heatmaps for gene expression were generated in GraphPad Prism 8 statistical software.

## 5. Conclusions

In the present study, it was shown that the application of SSE under both unstressed and NaCl-stressed conditions improved seedling growth and biomass parameters. In the same way, with the application of SSE and stress by NaCl, the concentration of photosynthetic pigments, nonenzymatic antioxidants, and proline, and the activity of antioxidant enzymes increased. Regarding the defense genes, stress increased their expression, and the use of SSE potentiated gene expression. This indicates that SSE can be an alternative for mitigating the negative effects of salt stress in tomato crops by inducing growth and activating the antioxidant system. However, SSE could also be used in other crops to improve yields and product quality, under both normal and stressful conditions.

## Figures and Tables

**Table 1:** Growth parameters of tomato seedlings.

Evaluation	Treatment	Seedling Height (cm)	Stem Diameter (mm)	Number of Leaves
	AC	12.00 ± 0.73 bc	3.18 ± 0.23 bc	6.00 ± 0.00 b
	ANCP	12.66 ± 1.05 b	3.22 ± 0.27 b	6.60 ± 0.55 b
11 DAT	SSE	14.04 ± 1.43 a	3.54 ± 0.32 a	7.40 ± 0.89 a
	AC + NaCl	11.18 ± 0.60 c	2.71 ± 0.14 d	6.00 ± 0.71 b
	ANCP + NaCl	11.50 ± 0.94 bc	2.91 ± 0.15 cd	6.20 ± 0.45 b
	SSE + NaCl	11.72 ± 0.91 bc	3.11 ± 0.18 bc	6.40 ± 0.55 b
	AC	24.02 ± 0.84 a	3.81 ± 0.15 bc	8.20 ± 0.45 c
	ANCP	25.40 ± 1.91 a	4.05 ± 0.25 ab	8.80 ± 0.45 b
21 DAT	SSE	26.20 ± 2.79 a	4.28 ± 0.30 a	9.60 ± 0.55 a
	AC + NaCl	19.18 ± 1.27 b	3.09 ± 0.14 d	7.80 ± 0.45 c
	ANCP + NaCl	20.78 ± 1.71 b	3.16 ± 0.18 d	8.00 ± 0.00 c
	SSE + NaCl	20.97 ± 0.67 b	3.55 ± 0.16 c	8.00 ± 0.00 c
	AC	35.60 ± 0.45 b	4.51 ± 0.15 c	10.20 ± 0.45 b
	ANCP	36.80 ± 2.68 ab	4.83 ± 0.35 b	11.00 ± 0.71 a
31 DAT	SSE	38.38 ± 2.62 a	5.18 ± 0.30 a	11.40 ± 0.55 a
	AC + NaCl	24.66 ± 1.09 c	3.49 ± 0.14 e	8.80 ± 0.45 c
	ANCP + NaCl	26.00 ± 0.60 c	3.56 ± 0.18 e	9.00 ± 0.00 c
	SSE + NaCl	26.44 ± 1.26 c	4.05 ± 0.16 d	9.20 ± 0.45 c

Different letters within each column indicate significant differences between treatments (LSD, *p* ≤ 0.05). DAT: Days after transplant; AC: Absolute control; ANCP: *A. nodosum* commercial product; SSE: *Sargassum* spp. seaweed extracts; NaCl: 100 mM sodium chloride; *n* = 5; ± standard deviation (SD).

**Table 2:** Fresh and dry biomasses of tomato seedlings at the end of the experiment.

Treatment	FAB	FRB	TFB	DAB	DRB	TDB
	(g Plant^−1^)	(g Plant^−1^)	(g Plant^−1^)	(g Plant^−1^)	(g Plant^−1^)	(g Plant^−1^)
AC	19.58 ± 1.34 b	3.95 ± 2.24 a–c	23.53 ± 3.14 b	3.52 ± 0.14 b	0.84 ± 0.09 b	4.36 ± 0.21 b
ANCP	20.49 ± 2.43 b	5.91 ± 3.55 a	26.40 ± 5.54 ab	3.69 ± 0.44 b	0.90 ± 0.13 b	4.59 ± 0.57 b
SSE	23.02 ± 1.51 a	5.04 ± 1.71 ab	28.06 ± 3.10 a	4.03 ± 0.33 a	1.12 ± 0.17 a	5.15 ± 0.43 a
AC + NaCl	9.32 ± 0.93 c	1.04 ± 0.57 d	10.36 ± 1.48 c	1.55 ± 0.14 c	0.37 ± 0.09 d	1.92 ± 0.23 c
ANCP + NaCl	10.04 ± 0.85 c	2.22 ± 1.41 cd	12.27 ± 2.23 c	1.61 ± 0.15 c	0.43 ± 0.07 cd	2.04 ± 0.21 c
SSE + NaCl	11.06 ± 1.11 c	2.43 ± 1.35 b–d	13.49 ± 2.02 c	1.72 ± 0.10 c	0.56 ± 0.15 c	2.28 ± 0.20 c
CV (%)	9.37	59.09	16.82	9.36	17.43	10

Different letters within each column indicate significant differences between treatments (LSD, *p* ≤ 0.05). AC: Absolute control; ANCP: *A. nodosum* commercial product; SSE: *Sargassum* spp. seaweed extracts; NaCl: 100 mM sodium chloride; FAB: Fresh aerial biomass; FRB: Fresh root biomass; TFB: Total fresh biomass; DAB: Dry aerial biomass; DRB: Dry root biomass; TDB: Total dry biomass; CV: Coefficient of variation; *n* = 5; ± SD.

**Table 3:** Biochemical characterization of SSE.

Composition	Concentration
pH	5.60 ± 0.10
EC (dS m^−1^)	0.83 ± 0.01
Total proteins (mg g^−1^ DW)	3.47 ± 0.08
GSH (mg g^−1^ DW)	3.29 ± 0.02
Amino acids (mg g^−1^ DW)	0.43 ± 0.008
Total phenols (mg EAG g^−1^ DW)	8.43 ± 0.79
Flavonoids (mg EC g^−1^ DW)	2.83 ± 0.04
IAA (mg kg^−1^ DW)	0.57 ± 0.07
tZ (µg g^−1^ DW)	175.99 ± 7.49
Glucose (mg 100 g^−1^ DW)	107.87 ± 0.004
Galactose (mg 100 g^−1^ DW)	74.01 ± 0.49
Fucose (mg 100 g^−1^ DW)	258.37 ± 9.82
Mannitol (mg 100 g^−1^ DW)	29.96 ± 0.40
ACTE DPPH (mg g^−1^ DW)	53.54 ± 1.70

SSE: *Sargassum* spp. seaweed extracts; EC: Electric conductivity; GSH: Reduced glutathione; IAA: Indole-3-acetic acid; tZ: Trans-zeatin; ACTE: Antioxidant capacity in trolox equivalents; DPPH: 2,2-diphenyl-1-picrylhydrazyl; DW: Dry weight; *n* = 3 except GSH, amino acids and total phenols (*n* = 5) ± SD.

**Table 4:** Primer sequences of the analyzed genes.

Gene	Forward Primer 5′-3′	Reverse Primer 5′-3′
*ACT*	CCCAGGCACACAGGTGTTA	CAGGAGCAACTCGAAGCTC
*NCED1*	CTTATTTGGCTATCGCTGAACC	CCTCCAACTTCAAACTCATTGC
*HSP70*	TGCTGGAGGTGTTATGACCA	GACTCCTCTTGGTGCTGGAG
*PIP2*	CTGCACCGTTGCTCGATTTT	GCGACAGTGACGTAGAGGAA
*P5CS1*	CTGTTGTGGCTCGAGCTGAT	GACGACCAACACCTACAGCA
*ERD15*	AGGCATCAAGTCATCACTCTCTGGT	GAGGTAAATGTGAGTAAGAACCAACG
*Fe-SOD*	CTGGGAATCTATGAAGCCCAACGGA	CAAATTGTGTTGCTGCAGCTGCCTT
*CAT1*	TCGCGATGGTGCTATGAACA	CTCCCCTGCCTGTTTGAAGT
*cAPX2*	GTGACCACTTGAGGGACGTGTTTGT	ACCAGAACGCTCCTTGTGGCATCTT
*PAL5-3*	GGAGGAGAATTTGAAGAATGCTGTG	TCCCTTTCCACCACTTGTAGC

## Data Availability

Not applicable.
